# Effects of Dietary Pantothenic Acid on Growth, Intestinal Function, Anti-Oxidative Status and Fatty Acids Synthesis of Juvenile Blunt Snout Bream *Megalobrama amblycephala*

**DOI:** 10.1371/journal.pone.0119518

**Published:** 2015-03-17

**Authors:** Yu Qian, Xiang-Fei Li, Ding-Dong Zhang, Dong-Sen Cai, Hong-Yan Tian, Wen-Bin Liu

**Affiliations:** College of Animal Science and Technology, Nanjing Agricultural University, Nanjing, Jiangsu Province, People’s Republic of China; CINVESTAV-IPN, MEXICO

## Abstract

Four groups of juvenile *Megalobrama amblycephala* were fed three times daily with six semi-purified diets containing 3.39 (PA unsupplied diet), 10.54, 19.28, 31.04, 48.38 and 59.72 mg kg^-1^ calcium D-pantothenate. The results showed that survival rate, final weight, specific growth rate, protein efficiency ratio and nitrogen retention efficiency all increased significantly (*P*<0.01) as dietary PA levels increased from 3.39 to 19.28 mg kg^-1^, whereas the opposite was true for feed conversion ratio. Whole-body crude protein increased as dietary PA levels increased, while the opposite pattern was found for the crude lipid content. Intestinal α-amylase, lipase, protease, Na^+^-K^+^-ATPase, alkaline phosphatase and gamma-glutamyl transferase activities were all elevated in fish fed PA-supplemented diets. Hepatic catalase activities improved with increases in dietary PA, while the opposite was true for malondialdehyde contents. The liver PA concentration and coenzyme A content rose significantly (*P*<0.01), up to 31.04 mg kg^-1^, with increasing dietary PA levels and then plateaued. The percentage of hepatic saturated fatty acids increased significantly (*P*<0.01) as dietary PA levels increased, while the percentages of monounsaturated fatty acids and polyunsaturated fatty acid (PUFA) decreased as dietary PA increased. Fish fed diets containing 19.28 and 31.04 mg kg^-1^ PA exhibited higher (*P*<0.01) docosahexaenoic acid and PUFA percentages in muscle than those fed with other diets. The expression of the gene encoding pantothenate kinase was significantly up-regulated (*P*<0.01) in fish fed PA-supplemented diets. Hepatic Acetyl-CoA carboxylase α, fatty acid synthetase, stearoyl regulatory element-binding protein 1 and X receptor α genes all increased significantly (*P*<0.01) as dietary PA levels increased from 3.39 to 31.04 mg kg^-1^. Based on broken-line regression analyses of weight gain, liver CoA concentrations and PA contents against dietary PA levels, the optimal dietary PA requirements of juvenile blunt snout bream were estimated to be 24.08 mg kg^-1^.

## Introduction

Pantothenic acid (PA) is an essential water-soluble vitamin for fish. It participates in numerous intermediary metabolic reactions as a component of coenzyme A (CoA), which plays a crucial role in reactions which glucose, fatty acids and amino acids enter into energy-yielding tricarboxylic acid cycle, acetylation of choline to from the neurotransmitter acetylcholine and biosynthesis of fatty acids [1–3]. Considering its significant role in intermediary metabolism, the optimal dietary PA requirement has been determined for several fish species during the past few decades, including common carp (*Cyprinus carpio* L.), blue tilapia (*Oreochromis aureus*), channel catfish (*Ictalurus punctatus*), rainbow trout (*Oncorhynchus mykiss*), grouper (*Epinephelus malabaricus*), Jian carp (*Cyprinus carpio var*. Jian) and yellowtail (*Seriola quinqueradiata*) [2–12]. However, the aforementioned studies have mainly focused on carnivorous and omnivorous fish, the optimal dietary PA requirement of herbivorous species has while received little attention. In addition, the optimal PA requirement for fish has generally been determined based on growth performance. The potential mechanisms underlying these progresses are still unknown. The growth of fish has been reported to be positively correlated with feed utilization, which depends greatly on the digestive and absorptive capacities [13]. It is generally acknowledged that the intestinal enzymes activities are reliable indicators of intestinal functions [14,15]. Accordingly, the investigations of intestinal enzymes activities might partly shed light on the growth retardation and poor feed efficiency of fish fed PA deficient diets. Unfortunately, the correlation between dietary PA levels and the digestive and absorptive capacities of fish still remains poorly understood.

So far, the effects of nutritional factors on the anti-oxidative defenses of fish have been extensively investigated. However, whether or not dietary PA supplementation could affect the oxidative status of fish is still unknown. Recently, PA and its related compounds have been reported to protect the cell membrane against damage caused by lipid peroxidation in an in vitro study using tumor cells [16], indicating the potential correlation between dietary PA levels and body anti-oxidative capability. In addition, it has been shown that oxidative stresses might render fish susceptible to different diseases [17]. This might suggest a close connection between oxidative status and health status of fish, as is still poorly understood. Therefore, oxidative status should also be considered when evaluating the optimal PA requirement for fish under farming conditions to assess its welfare state.

Previous studies indicated that dietary PA deficiency results in hepatic fat deposition of fish and results in the dysfunction of lipid metabolism [3,11]. However, whether this was resulted from an enhanced fatty acid synthesis or not is still unknown. As an essential vitamin for fish, PA served as the precursors of CoA and also donates a 4′-phosphopantetheine moiety to acyl carrier protein (ACP) [4]. Both CoA and ACP are important cofactors in body fatty acid synthesis, supplying precursors for condensation reactions and carrying the growing acyl chain from one enzyme to another [18]. The in vivo de novo fatty acid synthesis pathway is complex, involving the cooperation of many enzymes, coenzymes and chemical substances. Among these co-factors, Acetyl-CoA carboxylase α (ACCα) is a major regulatory enzyme, catalyzing the conversion of acetyl-CoA to malonyl-CoA, as is the first step of fatty acid biosynthesis [19]. In addition, the conversion of acetyl- and malonyl-CoA to long-chain saturated fatty acids is a key procedure in the FA biosynthetic pathway catalyzed by fatty acid synthetase (FAS) [20,21]. Moreover, sterol regulatory element-binding protein 1 (SREBP1) directly activates the expression of FAS, while liver X receptor α (LXRα) can enhance fatty acid synthesis through the induction of SREBP1 [22]. Considering their biological functions in fatty acid biosynthesis, the investigations of the expressions of these genes might give us some clues on how dietary PA influences the lipid metabolism of fish.

Bearing these in mind, the present study was conducted to estimate the optimal dietary PA requirement of juvenile blunt snout bream (*Megalobrama amblycephala*), an economically important herbivorous freshwater fish widely cultured in China. In addition, the potential regulatory effects of dietary PA on the intestinal enzymes activities, anti-oxidative status and fatty acid synthesis of fish were also investigated.

## Materials and Methods

### Ethic statement

Animal care and use were conducted in accordance with the Animal Research Institute Committee guidelines of Nanjing Agriculture University, China. This study was specifically approved by the Committee of the Animal Research Institute of Nanjing Agriculture University, China.

### Diets

The formulation and proximate composition of the basal diet are presented in Table 1, which contained 30.75% crude protein and 6.14% crude lipid [23]. Six semi-purified diets were formulated to contain 0, 8, 16, 32, 48 and 64 mg kg^-1^ calcium D-pantothenate. Fish meal, casein and gluten were adopted as protein sources. Equal proportions of fish oil and soybean oil were supplemented as lipid sources. Corn starch served as the single carbohydrate source. The PA concentrations of the experimental diets were determined to be 3.39, 10.54, 19.28, 31.04, 48.38 and 59.72 mg kg^-1^, respectively, via high-performance liquid chromatography (HPLC) [24].

**Table 1:** Formulation and proximate composition (% air-dry basis) of the basal diet.

Ingredients	%	Proximate composition	%
Fish meal	10.50	Crude protein	30.75
Casein	24.00	Crude lipid	6.14
Gelatin	6.00	Crude fiber	14.38
Corn starch	38.30	Crude ash	3.11
Fish oil	3.10	Gross energy (MJ kg^-1^)	16.16
Soybean oil	3.10		
α—Cellulose	10.00		
Premix without PA	1.20		
Calcium biphosphate	1.80		
Carboxymethylcellulose	2.00		
Total	100.00		

Premix provided the following minerals and/or vitamins (per kg premix): CuSO_4_·5H_2_O 2.00 g, FeSO_4_·7H_2_O 25.00 g, ZnSO_4_·7H_2_O 22.00 g, MnSO_4_·4H_2_O 7.00 g, Na_2_SeO_3_ 0.04 g, KI 0.026 g, CoCl_2_·6H_2_O 0.10 g, Vitamin A 900,000.00 IU, Vitamin D 200,000.00 IU, Vitamin E 4500.00 mg, Vitamin K3 220.00 mg, Vitamin B1 320.00 mg, Vitamin B2 1090.00 mg, Vitamin B6 500.00 mg, Vitamin B12 1.60 mg, Vitamin C 10,000.00 mg, Folic acid 165.00 mg, Choline 120,000.00 mg, Niacin 2500.00 mg, Biotin 100.00 mg, Myoinositol 15,000.00 mg.

All diets were prepared in the laboratory as detailed by Li *et al*. and Jiang *et al*. [25,26]. The ingredients were ground into fine powder and mixed thoroughly with soybean oil and fish oil until they were homogenous. Then, an appropriate amount of water was added to produce a stiff dough. The dough was subsequently pelleted using a laboratory pellet machine and dried in a ventilated oven at room temperature. The prepared feed was stored at -20°C in plastic-lined bags until use.

### Fish and feeding trial

Blunt snout bream juveniles were obtained from the Fish Hatchery of Yangzhou (Jiangsu, China). The feeding trial was performed in an indoor recirculating aquaculture system. Prior to the experiment, the fish were reared in several plastic tanks (3×0.8×0.8m, L:W:H) for 2 weeks to acclimate to the experimental conditions by feeding a commercial diet containing 320 g kg^-1^ crude protein and 60 g kg^-1^ crude lipid. After the conditioning period, fish of similar sizes (average initial weight of 6.04±0.02 g) were randomly distributed into 24 plastic tanks (3×0.8×0.8m, L:W:H) at a rate of 30 fish per tank. The fish in each aquarium were randomly assigned to one of six experimental diets. Each diet was tested in four replicates. The fish were fed to apparent satiation three times daily (8:00, 12:00 and 16:00 h) for 8 weeks. At each feeding time, we checked all of the tanks. If a dead fish was found, it would be weighed, recorded and stored at -20°C. No humane endpoints were used during the survival study. A 12:12 h light: dark regime (07:30–19:30 h, light period) was maintained with timed fluorescent lighting. The water temperature ranged from 25 to 30°C; pH fluctuated between 7.2 and 7.4; dissolved oxygen was maintained above 5.0 mg L^-1^; and total ammonia nitrogen and nitrite were kept below 0.2 and 0.005 mg L^-1^, respectively, during the feeding trial.

### Sample collection

Before harvest, the juvenile blunt snout bream were starved for 24 hours to ensure gastric emptiness. Then, the fish were anesthetized in diluted MS-222 at a concentration of 100 mg L^-1^. The total number and weight of the fish in each aquarium were subsequently determined. Samples of 20 fish at the beginning and 6 fish per tank at the end of the feeding trial were collected and stored at -20°C for body composition analysis. A total of 8 fish per tank were sacrificed for collection of liver and intestinal samples. Livers were quickly removed and stored at -20°C for subsequent analysis. Four of the livers were used for the measurement of anti-oxidative status and the concentrations of CoA and PA, while the others were analyzed for lipid and fatty acids contents. The intestine was also separated and scoured with physiological saline, then stored at -20°C for analysis of intestinal enzyme activities. The remaining fish were all sampled for the analysis of biometric parameters.

### Proximate composition analysis

The diets and fish were analyzed to determine their proximate composition according to the procedures detailed by the AOAC (1990) [27]. Moisture contents were determined by drying to a constant weight at 105°C. Crude protein (nitrogen×6.25) was measured using a Kjeltec Analyzer Unit. Crude lipid was determined via ether extraction using a Soxtec Auto Extraction Unit. Ash contents were measured through combustion at 550°C for 4 h. Liver PA contents were determined as described by Woollard *et al*. [24].

### Measurement of intestinal enzyme activities

Intestinal samples were carefully homogenized on ice in 10 volumes (w:v) of ice-cold physiological saline (0.85% (w:v)) and then centrifuged at 3, 000 rmp min^-1^ for 10 min at 4°C. The supernatant was subsequently analyzed to determine the activities of intestinal enzymes. Protein concentrations were determined with Folin-phenol reagent, using BSA as standard to enable the calculation of enzyme-specific activities [25,28]. Protease activity was assayed with 10 g kg^-1^ casein as substrate at the optimal pH for intestine (o.1 M Tris-HCl buffer, pH 8.0), 28°C for 45 min, stopped with 15% trichloroacetic acid (TCA), and the optical density of the supernatant was read at 280 nm against tyrosine as standard. A substrat-free control and an enzyme-free control were both run with the experimental samples. Specific activity was expressed as micromoles of hydrolyzed substrate min^-1^ g^-1^ tissue protein (U g^-1^ tissue protein) [29]. The activities of α-amylase, lipase,Na+,K+-ATPase, alkline phosphatase (AKP) and gamma-glutamyl transferase (γ-GT) were measured by the commercial kits [14, 30–33].

### Analysis of the hepatic anti-oxidative status

For determination of the hepatic anti-oxidative status, liver samples were prepared as described by Lygren *et al*. [34]. Briefly, the liver samples were homogenized on ice in 5 volumes (w:v) of ice-cold physiological saline 0.85% (w:v), followed by centrifugation at 3000 rmp min^-1^ for 10 min at 4°C. The supernatant was used for subsequent analysis. The liver malondialdehyde (MDA) content was determined using the thiobarbituric acid test according to Satho [35]. Liver superoxide dismutase (SOD), catalase (CAT) and glutathione peroxidase (GPX) activities as well as glutathione (GSH) concentrations were all determined following the methods described by Lygren *et al*. [34]. All these measurement was operated by the commercial kits.

### Tissue lipid concentration and fatty acid composition

The total lipid concentration in the liver was determined as described by Folch *et al*. using chloroform:methanol (2:1, v:v) to extract total lipids [36]. The fatty acids of the lipids were then methylated using 0.5 mol L^-1^ NaOH in methanol for 30 min at 60°C and esterified in 25% boron trifluoride (BF_3_) in methanol. FA methyl esters were subsequently analyzed and quantified using a Shimadzu GC-201 gas chromatograph in a cross-linked 5% phenyl methyl silicone gum phase column (length, 30 m; internal diameter, 0.32 mm; film thickness, 0.25 mm; N2 as the carrier gas), equipped with flame ionization detection. The injector and detector temperatures were both 250°C. The oven temperature was kept at 100°C for 3 min, then raised to 180°C at a rate of 10°C min^-1^ and to 240°C at a rate of 3°C min^-1^. The relative quantity of each FA was determined by measuring the area under the chromatograph peak.

### Total RNA extraction, reverse transcription and real-time PCR

Total RNA was extracted from the livers of juvenile blunt snout bream using the TRIzol reagent according to the manufacturer’s instructions and treated with RQ1 RNase-free DNase to eliminate genomic DNA contamination. The quantity and purity of the RNA was determined based on absorbance measurements (A260/280), and its integrity was tested via electrophoresis in 1.0% formaldehyde denaturing agarose gels. cDNA was generated from 500 ng of DNase-treated RNA using the ExScript RT-PCR kit. The mixture consisted of 500 ng of RNA, 2 μl of buffer (5×), 0.5 μl of a dNTP mixture (10 mM each), 0.25 μl of RNase inhibitor (40 U μl^-1^), 0.5 μl of a dT-AP primer (50 mM), 0.25 μl ExScript RTase (200 U μl^-1^) and 6.5 μl of DEPC H_2_O, in a total volume of 10 μl. The reaction conditions were as follows: 42°C for 40 min, 90°C for 2 min and 4°C thereafter. The resulting first-strand cDNA from each tissue was then diluted and used as a template for PCR. The set of primers for the PANKα, LXRα, ACCα, FAS and SREBP1 genes were designed with Primer 5 according to the codifying sequences obtained in our laboratory (Table 2). RT-PCR was conducted in a Mini Option real-time detector. The final volume of the amplification reactions was 25 μL, which contained 2 μL of a cDNA sample, 12.5 μL of 2×SYBR Green I Master Mix, 0.5 μL of each primer (Table 2) and 9.5 μL of dH2O. The RT-PCR protocol consisted of 3 min at 95°C, followed by 45 cycles of 10 s at 95°C and 20 s at 60°C. Melting curve analysis of the amplification products was performed at the end of each PCR run to confirm that the specific products were obtained. Each sample was run in triplicate. Reactions without the addition of the template were used as negative controls. At the end of the reaction, the obtained fluorescence data were converted into Ct values. The expression level of each gene was normalized to β-actin using the 2^−ΔΔCT^ method [37].

**Table 2:** Nucleotide sequences of the primers used to assay gene expression by real-time PCR.

Target gene	Forward (5′-3′)	Reverse (5′-3′)	Annealing temperature (°C)
coaA	TGGCTCGGGCGTCAGTA	GTCCATAGCATAGGCAAGAAG	53.50
ACCα	TCTGCCCTCTATCTGTCT	ATGCCAATCTCATTTCCT	52.50
FAS	GACCTGGAGGCTCGTGT	GGATGATGCCTGATGG	53.60
LXRα	ACGCCCTCCACTCTTACA	GCGGGAGTTTCTTGTCTT	52.00
SREBP1	GCTGGCGTGTCGCTATCT	TGTTGGCAGTCGTGGAGG	57.60
β-actin	TCGTCCACCGCAAATGCTTCTA	CCGTCACCTTCACCGTTCCAGT	52.00

coaA, pantothenate kinase gene; ACCα, acetyl-CoA carboxylase α gene; FAS, fatty acid synthase gene; LXRα, liver X receptor α; SREBP1, sterol regulatory element-binding protein-1

### Calculations

Surival rate (%) = final number of fish × 100 / initial fish number.

Specific growth rate (SGR, % day^-1^) = (Ln W_t_—Ln W_0_) × 100 / T.

Feed conversion ratio (FCR) = Feed consumption (g) / fish weight gain (g).

Protein efficiency ratio (PER) = Fish weight gain (g) / protein intake (g).

Dressout percentage (DP, %) = Carcass (with head and viscera removed) weight (g) × 100 / body weight (g).

Condition factor (CF, %) = Body weight (g) × 100 / body length (cm)^3^.

Hepatosomatic index (HSI, %) = Liver weight (g) × 100 / body weight (g).

Viscera / body ratio (VBR, %) = Viscera weight (g) × 100 / body weight (g).

Nitrogen retention efficiency (NRE, %) = [(W_t_ × C_t_)—(W_0_ × C_0_)] × 100 / (C_diet_ × feed intake (g)), where W_0_ and W_t_ are the initial and final body weight; T is the culture period in days; C_0_ and C_t_ are the initial and final nitrogen contents in whole body, respectively; and C_diet_ is the nitrogen content in the diets.

### Statistical analysis

The data were subjected to one-way analysis of variance (ANOVA) to test the effects of dietary PA levels on the performance of fish after testing the homogeneity of variances with the Levene test. When significant (*P*<0.05) differences were found, Duncan’s multiple range test was used to rank the means. Percentage data were arc-sine transformed prior to ANOVA and reversed afterward [38]. The analyses were performed using the SPSS program, version 16.0 for Windows. All data are presented as the means ± SD (standard deviation) of four replicates. In addition, broken-line regression analysis was performed to determine the regression of weight gain, liver PA contents and the hepatic CoA concentration on dietary PA levels to establish the optimum PA requirements [39].

## Results

### Growth performance and feed utilization

In the 6^th^ week of the feeding trail, the blunt snout bream juveniles fed the control diet began to show signs of deficiency, such as anorexia, abnormal swimming activity, growth retardation and hemorrhage of the body surface and fins.

**Fig 1:**
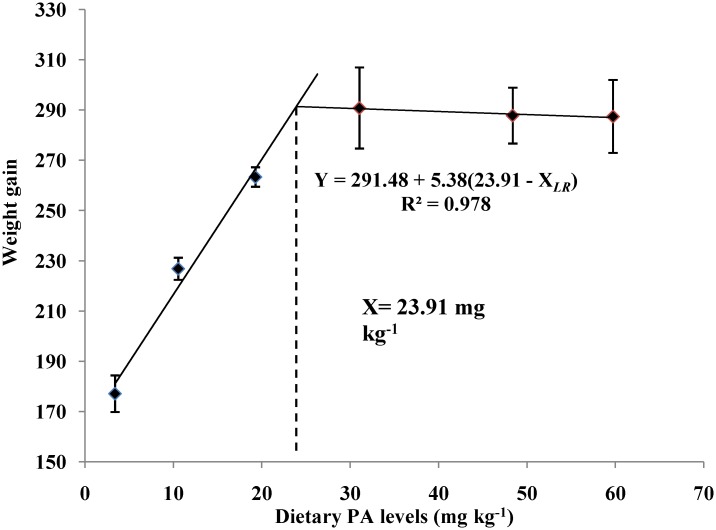
Relationship between dietary PA levels and weight gain (%) of juvenile blunt snout bream.

The growth performance and feed utilization of the juvenile blunt snout bream are presented in Table 3. The survival rate, final weight, SGR, PER and NRE of the juvenile blunt snout bream all increased significantly (*P*<0.01) as dietary PA levels increased from 3.39 to 31.04 mg kg^-1^, whereas the opposite was true for the FCR. However, these parameters all showed no additional significant changes (*P*>0.05) with further increases in PA levels. Based on the broken-line regression analysis of weight gain against dietary PA levels, the optimal dietary PA level for juvenile blunt snout bream was estimated to be 23.91 mg kg^-1^ (Fig. 1).

**Table 3:** Effects of dietary PA levels on growth performance and feed utilization of juvenile blunt snout bream.

PA levels (mg kg^-1^)	Survival rate (%)	Initial weight (g)	Final weight (g)	SGR (%)	FCR	PER	NRE (%)
3.39	78.33±3.19^a^	6.04±0.03	16.74±0.51^a^	1.82±0.05^a^	3.52±0.11^c^	1.18±0.07^a^	17.95±1.17^a^
10.54	93.33±2.72^b^	6.04±0.04	19.74±0.28^b^	2.11±0.02^b^	2.81±0.25^b^	1.26±0.09^ab^	19.13±1.26^ab^
19.28	95.00±3.19^b^	6.06±0.02	22.01±0.25^c^	2.30±0.02^c^	2.76±0.25^ab^	1.26±0.09^ab^	21.22±1.99^abc^
31.04	96.67±1.92^b^	6.04±0.04	23.62±1.01^c^	2.43±0.08^c^	2.21±0.17^a^	1.53±0.09^c^	23.74±1.00^c^
48.38	95.00±1.67^b^	6.05±0.05	23.48±0.76^c^	2.42±0.05^c^	2.27±0.09^ab^	1.50±0.04^bc^	22.96±1.03^bc^
59.72	96.67±1.92^b^	6.04±0.05	23.39±0.84^c^	2.42±0.06^c^	2.49±0.13^ab^	1.36±0.10^abc^	20.69±1.41^abc^

Values are presented as mean ± SD of four replications (n = 4). Means in the same column with different superscripts are significantly different (*P* < 0.05). SGR, specific growth rate; FCR, feed conversion ratio; PER, protein efficiency ratio; NRE, nitrogen retention efficiency.

As can be seen from Table 4, CF increased significantly (*P*<0.01) as dietary PA levels increased from 3.39 to 19.28 mg kg^-1^, though no additional significant differences (*P*>0.05) were found in the groups with further increases in PA levels. However, DP, HSI and VBR displayed no significant differences (*P*>0.05) among all of the treatments.

**Table 4:** Effects of dietary PA levels on body parameters of juvenile blunt snout bream.

PA levels (mg kg^-1^)	DP (%)	CF (%)	HSI (%)	VBR (%)
3.39	60.18±1.54	1.82±0.02^a^	2.47±0.22	10.52±0.37
10.54	60.59±0.98	1.89±0.07^a^	2.58±0.30	10.94±0.95
19.28	60.93±0.38	1.97±0.02^b^	2.54±0.04	11.20±0.29
31.04	60.93±1.27	1.99±0.05^b^	2.54±0.05	11.51±0.34
48.38	61.56±1.52	2.00±0.01^b^	2.26±0.13	11.26±0.50
59.72	60.87±0.93	1.99±0.05^b^	2.48±0.08	11.41±0.11

Values are presented as mean ± SD of four replications (n = 4). Means in the same column with different superscripts are significantly different (*P* < 0.05). DP, dressout percentage; CF, condition factor; HSI, hepatosomatic index.

### Body composition and liver lipid content

The details presented in Table 5 show that the graded dietary PA levels had no significant (*P*>0.05) effects on body ash and moisture contents. The body lipid content decreased significantly (*P*<0.01) as dietary PA levels increased from 3.39 to 31.04 mg kg^-1^, though no additional significant differences were found (*P*>0.05) with further increases in dietary PA levels, whereas the crude protein content exhibited an opposite trend. The liver lipid contents of fish fed 3.39 and 10.54 mg kg^-1^ PA were significantly (*P*<0.01) higher than those of the other groups.

**Table 5:** Effects of dietary PA levels on whole-body composition (% wet weight) and liver lipid content (% wet liver weight) of juvenile blunt snout bream.

PA levels (mg kg^-1^)	Moisture (%)	Ash (%)	Crude protein (%)	Crude lipid (%)	Liver Lipid (%)
3.39	76.17±0.25	3.41±0.06	12.86±0.32^a^	6.59±0.44^d^	9.59±0.26^b^
10.54	75.91±0.08	3.44±0.05	13.76±0.17^b^	5.94±0.15^cd^	8.10±0.57^ab^
19.28	75.80±0.24	3.55±0.11	14.37±0.28^bc^	5.53±0.13^bc^	7.35±0.60^a^
31.04	75.85±0.21	3.48±0.10	14.67±0.13^cd^	4.58±0.31^a^	7.82±0.45^a^
48.38	75.66±0.20	3.43±0.11	14.44±0.33^c^	4.65±0.31^a^	7.36±0.65^a^
59.72	75.86±0.35	3.58±0.08	14.60±0.75^c^	4.73±0.18^ab^	7.80±0.55^a^

Values are presented as mean ± SD of four replications (n = 4). Means in the same column with different superscripts are significantly different (*P* < 0.05).

### Liver CoA and PA contents

**Fig 2:**
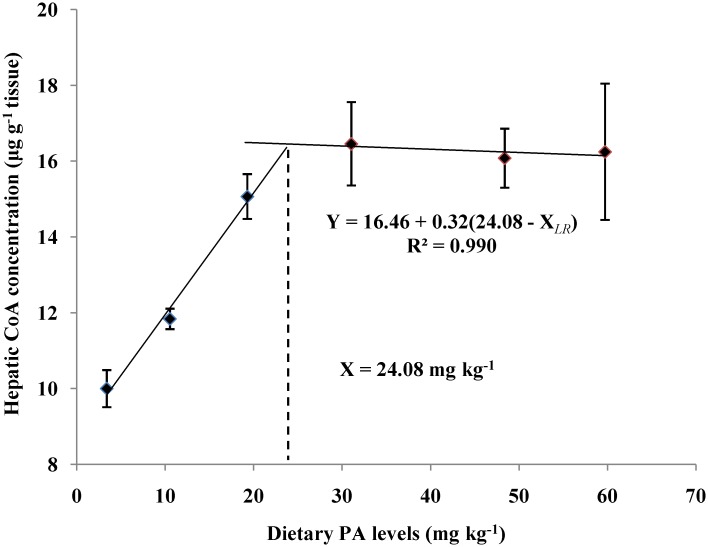
Relationship between dietary PA levels and hepatic PA concentration (μg g^-1^ tissue) of juvenile blunt snout bream.

**Fig 3:**
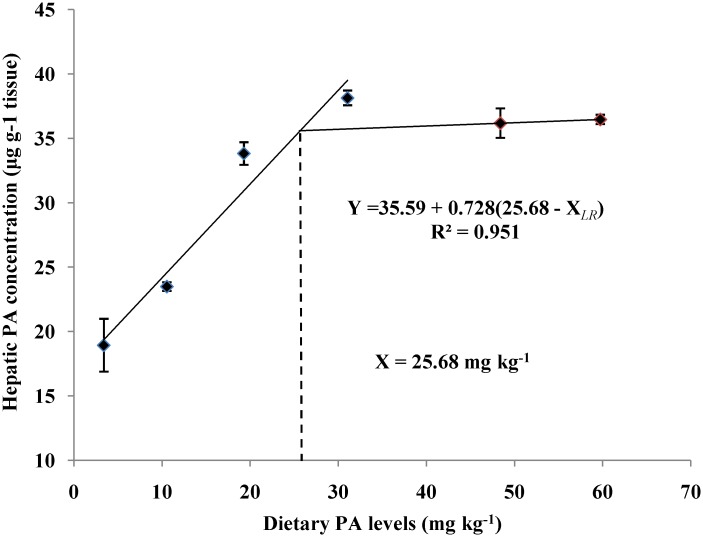
Relationship between dietary PA levels and hepatic CoA concentration (μg g^-1^ tissue) of juvenile blunt snout bream.

The liver CoA and PA contents of juvenile blunt snout bream fed different dietary PA levels was shown in Fig. 2 and Fig. 3. The liver CoA concentration increased significantly (*P*<0.01) as dietary PA levels increased from 3.39 to 19.28 mg kg^-1^ but showed no additional significant differences (*P*>0.05) with further increases in dietary PA levels. The liver PA concentration also rose significantly as dietary PA levels increased from 3.39 to 19.28 mg kg^-1^, then displayed a plateau up to 59.72 mg kg^-1^. Based on the broken-line regression analysis of liver CoA and PA concentrations against dietary PA levels, the optimal dietary PA levels for juvenile blunt snout bream were estimated to be 24.08 and 25.68 mg kg^-1^, respectively.

### Intestinal absorptive and digestive enzyme activities

The intestinal enzyme activities determined in the juvenile blunt snout bream fed different dietary PA levels are shown in Table 6. The activities of intestinal α-amylase, lipase, protease, Na^+^-K^+^-ATPase, AKP and γ-GT all increased significantly (*P*<0.01) as dietary PA levels increased from 3.39 to 10.54 mg kg^-1^. However, these parameters all showed no additional significant differences (*P*>0.05) with further increases in dietary PA levels.

**Table 6:** Effects of dietary PA levels on intestinal absorptive enzyme and digestive enzyme activities of juvenile blunt snout bream.

Dietary PA levels (mg kg^-1^)	Na^+^-K^+^-ATPase (U g^-1^ prot)	γ-GT (U g^-1^ prot)	AKP (U mg^-1^ prot)	Lipase (U g^-1^ prot)	α-Amylase (U g^-1^ prot)	Protease (U mg^-1^ prot)
3.39	5.33±0.59^a^	9.90±1.09^a^	35.34±3.06^a^	8.18±0.21^a^	713.38±28.80^a^	77.65±3.25^a^
10.54	9.35±0.97^b^	14.96±0.71^b^	48.36±0.79^b^	12.77±1.01^a^	824.39±15.36^b^	92.49±3.12^b^
19.28	11.26±0.78^bc^	16.82±0.44^bc^	87.31±1.19^d^	19.42±1.22^c^	819.41±6.18^b^	104.83±4.83^b^
31.04	11.48±0.83^bc^	16.50±0.24^bc^	72.83±1.12^c^	21.19±1.23^c^	857.61±16.36^b^	102.34±3.58^b^
48.38	12.74±1.24^c^	18.58±0.55^c^	82.07±4.27^d^	19.60±1.06^c^	832.94±6.91^b^	106.24±6.41^b^
59.72	11.96±0.84^bc^	17.71±0.60^c^	88.02±2.17^d^	20.91±2.77^c^	852.48±13.08^b^	105.27±6.99^b^

Values are presented as mean ± SD of four replications (n = 4). Means in the same row with different superscripts are significantly different (*P* < 0.05). γ-GT, gamma-glutamyl transferase; AKP, alkline phosphatase.

### Hepatic anti-oxidative status

As can be seen in Table 7, liver SOD and GPX activities as well as GSH contents showed little difference (*P*>0.05) among all of the treatments. The liver CAT activities of fish fed 31.04 and 48.38 mg kg^-1^ PA were significantly (*P*<0.01) higher than those of fish fed the control diet, but they exhibited no significant differences (*P*>0.05) from those of the other groups. In addition, liver MDA in fish fed the control diet was significantly (*P*<0.01) higher than those in the other groups.

**Table 7:** Effects of dietary PA levels on hepatic ant-oxidative status of juvenile blunt snout bream.

Dietary PA levels (mg kg^-1^)	MDA (nmol mg^-1^ prot)	CAT (U g^-1^ prot)	GSH (mg g^-1^ prot)	GPX (U mg^-1^ prot)	SOD (U g^-1^ prot)
3.39	12.40±0.32^b^	10.07±0.62^a^	4.36±0.51	41.44±8.99	64.85±4.75
10.54	8.57±0.33^a^	11.21±0.69^ab^	4.17±0.66	46.96±5.21	70.46±10.57
19.28	9.37±0.50^a^	10.67±0.85^a^	5.18±0.61	39.73±9.66	74.38±9.45
31.04	8.20±0.79^a^	14.80±1.23^b^	4.97±0.43	31.35±4.73	69.45±7.34
48.38	9.45±0.83^a^	14.67±1.43^b^	4.79±0.18	36.87±3.75	73.99±11.43
59.72	8.64±0.93^a^	13.94±1.90^ab^	4.96±0.22	33.87±3.39	68.09±3.34

Values are presented as mean ± SD of four replications (n = 4). Means in the same row with different superscripts are significantly different (*P* < 0.05). MDA, Malondialdehyde; CAT, catalase; GSH, glutathione; GPX, glutathione peroxidase; SOD, superoxide dismutase.

### Fatty acid composition

Effects of graded dietary PA levels on the FA composition of the liver and muscle are shown in Table 8, 9. In liver, the level of palmitic acid (16:0) increased significantly (*P*<0.01) when dietary PA levels were increased up to 19.28 mg kg^-1^. The variation of saturated fatty acids (SFA) showed a similar trend to palmitic acid, while the opposite was pattern was found for the levels of monounsaturated fatty acids (MUFA), docosahexaenoic acid (DHA) and polyunsaturated fatty acids (PUFA).

**Table 8:** Effects of dietary PA levels on liver fatty acids composition of juvenile blunt snout bream.

	Dietary PA levels					
	3.39	10.54	19.28	31.04	48.38	59.72
Fatty acid composition (% of total fatty acids)						
C14:0	1.66±0.02^bc^	1.76±0.03^c^	1.64±0.01^b^	1.38±0.01^a^	1.42±0.00^a^	1.36±0.08^a^
C16:0	19.82±0.75^a^	20.51±0.52^a^	23.97±0.77^b^	24.20±0.72^b^	24.15±0.41^b^	24.07±0.70^b^
C18:0	13.89±0.68	13.53±0.80	13.00±0.05	13.30±0.01	13.19±0.03	13.43±0.06
C20:0	0.13±0.00^b^	0.12±0.01^b^	0.13±0.01^b^	0.10±0.01^a^	0.13±0.01^b^	0.11±0.01^a^
ΣSFA	35.49±0.47^a^	35.93±1.27^a^	38.74±0.72^b^	38.98±0.71^b^	38.89±0.40^b^	38.98±0.62^b^
C16:1n-9	3.23±0.26^bc^	3.38±0.52^c^	3.26±0.06^bc^	3.38±0.03^c^	2.530±10^a^	2.78±0.08^ab^
C18:1n-9	31.34±0.50	31.41±0.76	30.92±0.16	30.03±0.30	30.70±1.71	30.02±0.69
C20:1n-9	0.88±0.03^b^	0.82±0.04^ab^	0.73±0.05^a^	0.75±0.05^a^	0.73±0.05^a^	0.78±0.02^ab^
ΣMUFA	35.44±0.34	35.61±0.96	34.91±0.24	34.15±0.30	33.96±0.99	33.57±0.78
C18:2n-6	13.17±0.04	13.15±0.64	12.10±0.60	13.13±0.30	13.50±0.54	13.19±0.34
C18:3n-3	0.89±0.11	0.88±0.13	0.82±0.02	0.77±0.03	0.94±0.03	0.83±0.20
C20:5n-3(EPA)	0.93±0.06	0.81±0.08	0.71±0.10	0.75±0.03	0.77±0.08	0.77±0.06
C22:5n-3	0.53±0.12	0.54±0.15	0.52±0.12	0.58±0.00	0.58±0.11	0.57±0.03
C22:6n-3(DHA)	6.34±0.82^b^	5.64±0.58^ab^	4.98±0.22^ab^	4.00±0.25^a^	3.97±0.50^a^	4.70±0.58^ab^
ΣPUFA	21.87±0.69^c^	21.02±0.51^bc^	19.14±0.46^a^	19.98±0.30^ab^	20.51±0.65^abc^	20.55±0.14^abc^
EPA+DHA	7.27±0.81^b^	6.45±0.62^ab^	5.69±0.28^ab^	5.50±0.28^a^	5.48±0.26^a^	5.96±0.53^ab^

Values are presented as mean ± SD of four replications (n = 4). Means in the same row with different superscripts are significantly different (*P* < 0.05).

**Table 9:** Effects of dietary PA levels on muscle fatty acids composition of juvenile blunt snout bream.

	Dietary PA levels					
	3.39	10.54	19.28	31.04	48.38	59.72
Fatty acid composition (% of total fatty acids)						
C14:0	1.63±0.06	1.80±0.08	1.75±0.06	1.68±0.01	1.82±0.07	1.69±0.07
C16:0	22.21±0.97	22.37±0.73	22.03±0.26	21.79±0.28	21.36±0.49	21.92±0.18
C18:0	7.49±0.54	7.49±0.62	8.07±0.37	7.84±0.02	7.87±0.23	8.25±0.14
C20:0	0.21±0.01	0.20±0.01	0.22±0.04	0.20±0.01	0.25±0.01	0.25±0.01
ΣSFA	31.54±0.63	31.86±0.36	32.06±0.16	31.51±0.29	31.31±0.28	32.11±0.06
C16:1n-9	3.26±0.09	3.83±0.23	3.33±0.08	3.28±0.06	3.49±0.21	3.44±0.58
C18:1n-9	28.60±0.94	28.90±0.27	27.44±0.39	28.40±0.48	27.99±0.48	27.89±0.64
C20:1n-9	0.58±0.00	0.53±0.06	0.54±0.02	0.57±0.02	0.59±0.08	0.59±0.08
ΣMUFA	32.43±1.01^ab^	33.27±0.43^b^	31.32±0.39^a^	32.25±0.52^ab^	32.06±0.58^ab^	31.92±0.25^ab^
C18:2n-6	17.52±0.11^cd^	16.50±0.32^a^	17.33±0.05^bc^	16.55±0.14^ab^	18.23±0.50^d^	17.94±0.18^cd^
C18:3n-3	1.16±0.05^a^	1.26±0.09^ab^	1.24±0.10^ab^	1.21±0.03^ab^	1.32±0.10^ab^	1.53±0.20^b^
C20:5n-3(EPA)	1.97±0.04	2.28±0.01	2.56±0.06	2.53±0.19	2.31±0.58	2.28±0.10
C22:5n-3	0.62±0.10^a^	0.94±0.02^b^	1.08±0.02^b^	1.07±0.03^b^	1.05±0.0.05^b^	1.01±0.04^b^
C22:6n-3(DHA)	8.15±0.38^a^	8.64±0.16^ab^	9.51±0.12^b^	9.81±0.37^b^	8.70±0.76^ab^	8.07±0.32^a^
ΣPUFA	29.42±0.23^a^	29.63±0.32^a^	31.73±0.13^b^	31.16±0.48^b^	31.61±0.68^b^	30.84±0.13^b^
EPA+DHA	10.12±0.42^a^	10.92±0.15^abc^	12.07±0.07^bc^	12.34±0.38^c^	11.01±1.24^abc^	10.36±0.33^ab^

Values are presented as mean ± SD of four replications (n = 4). Means in the same row with different superscripts are significantly different (*P* < 0.05).

No significant (*P*>0.05) difference was observed in SFA levels among all the treatments in muscle. The DHA levels increased markedly as dietary PA levels increased from 3.39 to 19.28 mg kg^-1^, but showed a slight decreasing trend with further increases in dietary PA levels. In addition, the muscle PUFA levels in fish fed 3.39 and 10.54 mg kg^-1^ PA were significantly lower (*P*<0.01) than those of the other groups. However, the levels of n-3 highly unsaturated fatty acids (DHA+EPA) increased significantly (*P*<0.01) with increasing dietary PA levels.

### Expression of the hepatic coaA, LXRα, ACC, FAS and SREBP1genes

**Fig 4:**
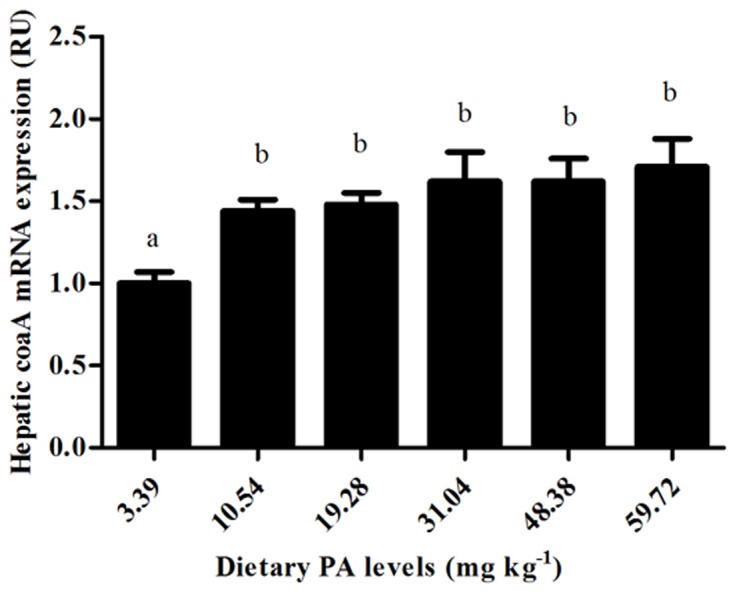
Relative mRNA expression of *coaA* gene in liver of juvenile blunt snout bream affected by dietary PA levels.

**Fig 5:**
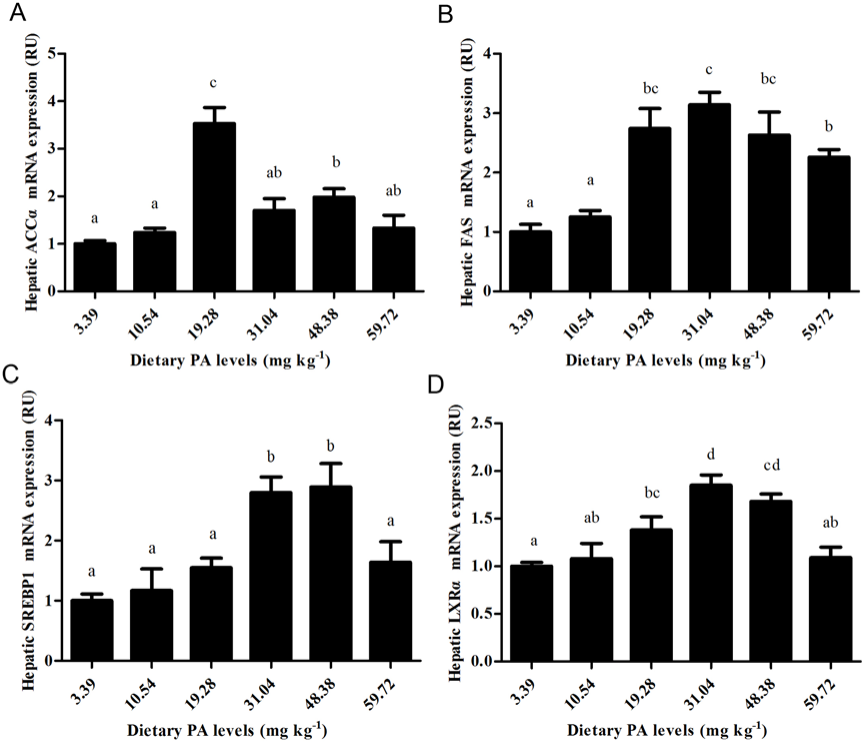
Relative expressions of fatty acid synthesis-related genes. (A) Relative ACCα gene expression affected by dietary PA levels. (B) Relative FAS gene expression affected by dietary PA levels. (C) Relative SREBP_1_ gene expression affected by dietary PA levels. (D) Relative LXRα gene expression affected by dietary PA levels.

The details presented in Fig. 4 and Fig. 5 show that graded dietary PA levels had significant (P<0.01) effects on the relative mRNA expression of the coaA, LXRα, ACCα, FAS and SREBP1genes in the liver of juvenile blunt snout bream. The expression of the coaA gene in fish fed the control diet was significantly (*P*<0.01) lower than in the other groups. The expression of LXRα, ACCα, FAS and SREBP1 all increased significantly (*P*<0.01) as dietary PA levels increased from 3.39 to 31.04 mg kg^-1^, then decreased slightly with further increases in dietary PA levels (*P*>0.05).

## Discussion

Dietary PA deficiency usually results in several deficiency syndromes in fish, including growth retardation and poor feed efficiency [3–11]. The results of this study clearly demonstrated that PA is essential for the normal growth of juvenile blunt snout bream, as was supported by the high mortality and poor growth performance observed both in fish fed the control diet. In addition, final weight and SGR both increased significantly with increasing dietary PA levels, while the opposite was true for FCR. According to previous studies, this growth improvement may be ascribed to the following factors. Firstly, dietary PA supplementation enhanced the tissue CoA concentration of juvenile blunt snout bream, as consequently promoted its growth performance. This is supported by the fact that CoA is generally recognized as the most important metabolic component of the intermediary metabolism [4], and a positive correlation between tissue CoA contents and body growth is generally observed in animals [40]. In fact, liver CoA content in this study increased significantly as dietary PA levels increased from 3.39 to 31.04 mg kg^-1^ and then plateaued, further confirming this assumption. Secondly, dietary PA supplementation improved the body protein deposition of juvenile blunt snout bream, as consequently benefited its growth performance. This is supported by the fact that protein deposition usually makes the greatest contribution to fish body growth [41]. In fact, in this study, weight gain, PER, NRE and whole-body protein content of fish all increased significantly as dietary PA levels increased from 3.39 to 31.04 mg kg^-1^, indicating that the ability of fish to utilize and deposit protein might be strengthened by dietary PA supplementation. Thirdly, dietary PA supplementation enhanced the intestinal digestive and absorptive enzymes activities of juvenile blunt snout bream, leading to the improved feed utilization and growth. This was supported by the fact that the growth of fish is often positively correlated with feed utilization, which depends greatly on the digestive and absorptive functions of intestine [15,42,43]. In fact, it has been reported in terrestrial animals that PA can maintain the integrity and the normal function of intestine [44]. In the present study, the lowest intestinal absorptive and digestive enzyme activities were all observed in the fish fed the control diet, indicating that PA deficiency may lead to the hampered intestinal function of blunt snout bream, as consequently resulting in growth retardation. Based on the broken-line regression analysis of weight gain against dietary PA levels, the optimal dietary PA level for juvenile blunt snout bream was estimated to be 23.91 mg kg^-1^. However, higher requirements were obtained by the regression analysis between dietary PA levels and hepatic PA and CoA concentrations, as was in line with the results observed in common carp, yellowtail and grouper [2,3,12]. Nevertheless, to achieve the rapid growth and maintain normal physiological functions, the optimal PA requirement of juvenile blunt snout bream may be 24.08 mg kg^-1^, which was based on the broken-line regression analysis of liver CoA content.

To date, the role of dietary PA on intestinal function has been widely investigated in mammals [45], and a consensus has been reached that intestinal function is positively correlated with tissue CoA content [46]. For example, the morphological changes and low transporting ability of intestine observed in pigs fed PA deficient diet have been ascribed to low tissue CoA content [44]. However, there is quite limited information concerning the effects of dietary PA levels on the intestinal functions of fish. In the present study, fish fed control diet obtained the lowest intestinal activities of Na^+^-K^+^-ATPase, γ-GT, AKP, protease, amylase and lipase, indicating that dietary PA deficiency might result in the hampered intestinal functions of blunt snout bream. This was supported by the fact that nutrient digestion and absorption at the intestinal epithelium is an ATP-bound process catalyzed by both intestinal brush border enzymes (such as Na^+^-K^+^-ATPase, γ-GT and AKP) and digestive enzymes (protease, amylase and lipase), whose activities can directly reveal the digestive and absorptive capacities of fish [15]. In fact, intestinal enzymes generally play a key role in the nutrient utilization of stomachless fish such as blunt snout bream. According to previous studies, this hypofunction might be ascribed to the abnormal energy metabolism since CoA is a crucial participant in the tricarboxylic acid cycle [47]. In addition, the activities of these intestinal enzymes all increased significantly as dietary PA increased from 3.39 to 10.54 mg kg^-1^ and then plateaued, indicating that dietary PA is essential for the maintenance of the normal intestinal functions of fish. The same trend was also observed in liver CoA content, as further confirmed the speculation that intestinal function is positively correlated with tissue CoA content.

In the present study, the lowest liver CAT activity was obtained in fish fed the control diet. Similar results were also found in liver SOD and GPX activities as well as GSH contents although no significant difference was observed. This indicated that dietary PA deficiency might cause oxidative stress of juvenile blunt snout bream, as was supported by the fact that the SOD-CAT system represents the first line of defense against oxidative stress and augmented activities of these free radical-scavenging enzymes (also include GPX and glutathione-S-transferase) usually indicate an enhanced anti-oxidative capability [48]. In addition, the highest liver MDA content was observed in fish fed PA-deficient diet, indicating again that dietary PA deficiency might cause oxidative stress of juvenile blunt snout bream. According to previous studies, tissue MDA levels provide a direct evidence of lipid peroxidation caused by free radicals or disordered lipid metabolism [49]. Furthermore, liver CAT activities increased while hepatic MDA contents decreased with increasing dietary PA levels, suggesting that optimal PA level could alleviate the cellular and/or molecular damages caused by oxidative stress [48,49]. The exact mechanisms underlying this progress are still unknown, since limited information is available concerning the correlations between dietary PA levels and the anti-oxidative status of fish. However, it might be related to the lipid dysmetabolism of fish. In this study, dietary PA deficiency results in both high liver lipid content and FUFA of juvenile blunt snout bream. And previous studies indicated that excess PUFA in liver are more likely to cause lipid peroxidation of blunt snout bream [50,51]. In addition, the protective effects of CoA might not be neglected since it not only prevents cell damage from lipid peroxidation, but also promotes cellular repair mechanisms through the potentiating synthesis of membrane phospholipids [16].

In the present study, hepatic CoA contents increased markedly as dietary PA levels increased from 3.39 to 19.28 mg kg^-1^. This might be ascribed to the enhanced expression of the pantothenate kinase gene (coaA). This was supported by the fact that coaA is a very important determinant of the CoA biosynthetic rate, since pantothenate kinase catalyzes the rate-controlling step in CoA biosynthesis [52]. In addition, compared to those fed control diet, fish fed PA-supplemented diets obtained relative high liver CoA content but a stable level of coaA expression in this study. This result was out of expectations since CoA biosynthesis is governed by the feedback inhibition of pantothenate kinase, which is further mediated by the concentration of intracellular nonesterified CoA [4,52,53]. This suggested that the regulation of coaA expression in blunt snout bream showed no direct relationship with the feedback inhibition of pantothenate kinase activities by CoA. Furthermore, in this study, the whole-body and liver lipid contents of juvenile blunt snout bream both decreased with increasing dietary PA supplementation, in line with the results observed in Jian carp, grouper and grass shrimp [3,11,54]. According to previous studies, the high body lipid content of fish fed the control diet might be ascribed to the low liver CoA content, as hepatic CoA concentration is generally positively correlated with body lipid transport [50,51]. In fact, fatty acids must first be activated by CoA before they can be synthesized into triglycerides [3]. Therefore, a low liver CoA content may inhibit the synthesis of triglycerides, as might consequently block lipid transport [51]. In addition, previous studies have found that both DHA and EPA can attenuate the synthesis and secretion of triglycerides in mammals [55]. In a recent study, triglycerides secretion was found to be inhibited by elevated percentages of DHA and EPA in the liver of blunt snout bream [51]. Therefore, the increased liver PUFA content of the fish fed the control diet in this study may be another cause of high body lipid accumulation.

In the present study, hepatic palmitic acid content and the SFA content both increased significantly as dietary PA levels increased from 3.39 to 19.28 mg kg^-1^, suggesting an enhanced synthesis of hepatic long-chain SFA by dietary PA supplementation. This is supported by the fact that palmitic acid (C16:0) is the major product of liver de novo fatty acid synthesis [20]. This increased palmitic acid content might be partially ascribed to the up-regulation of ACCα and FAS, as both genes play an important role in body fatty acid biosynthesis [56]. This is further supported by the fact that both hepatic ACCα and FAS expression showed a similar trend to palmitic acid content in this study. According to previous studies, the increased ACCα and FAS expressions might be a direct result of the up-regulation of SREBP1 expression, which in turn was activated by LXRα, since SREBP1 is an important regulatory factor activating genes required for the programming of fatty acid synthesis, while LXRα positively regulates the transcription of SREBP1[22]. In addition, it was interesting to find that fish fed PA-deficient diet exhibited lower expressions of ACCα, FAS SREBP1 and LXRα than the other groups, accompanied by a high liver PUFA content. This result is justifiable since fatty acid synthesis is subjected to the feedback inhibition of long-chain fatty acids (saturated or unsaturated) [21,57]. In this case, the LXRα-mediated regulation of SREBP-1c appears to be one mechanism by which unsaturated fatty acids suppress fatty acid synthesis through the suppression of SREBP-1c transcription [58]. In the present study, muscle PUFA levels showed an increasing trend with increasing dietary PA levels. According to previous studies, this might be attributed to the increased muscle ACP content observed in fish fed PA-supplemented diets, as the PUFA biosynthetic pathway is catalyzed by β-hydroxydecanoyl-ACP dehydrase which introduces a double bond into a growing fatty acid chain at the expense of β-hydroxydecanoyl-ACP [59]. In addition, in this study, the HUPA percentage in muscle was higher than that in liver, indicating that more PUFA accumulated in this tissue [51].

In summary, juvenile blunt snout bream requires exogenous PA to maintain normal growth and physiological functions. Based on the regression analyses between dietary PA levels and weight gain, liver CoA and PA contents, the optimal dietary PA requirements of this species were estimated to be 24.08 mg kg^-1^. In addition, PA deficiency caused oxidative stress and intestinal hypofunction which could be remitted by dietary PA supplementation. Furthermore, the expressions of various genes (including coaA, ACCα, LXRα and SREBP1) involved in liver fatty acid synthesis increased with increasing dietary PA levels, resulting in the enhanced liver SFA synthesis and increased muscle PUFA percentage.

## Supporting Information

S1 Checklist The ARRIVE Guidelines Checklist-Animal Research: Reporting In Vivo Experiments. (PDF)

S1 Dataset Segment sequences of *coaA*, ACCα, FAS, SREBP_1_ and LXRα genes. (XLSX)

S1 Editorial Certificate The editorial certificate of the manuscript. (PDF)
